# Supplementary material for: Structural anatomy of Protein Kinase C C1 domain interactions with diacylglycerol and other agonists
Source: Nat Commun. 2022 May 16;13:2695. doi: 10.1038/s41467-022-30389-2 (PMC9110374; doi:10.1038/s41467-022-30389-2)
Supplement: Supplementary file 1 — Supplementary Information [file 41467_2022_30389_MOESM1_ESM.pdf]

Supplementary Information

Structural anatomy of Protein Kinase C C1 domain interactions with diacylglycerol and other agonists

Sachin S. Katti[1], Inna V. Krieger[1], Jihyae Ann[2], Jeewoo Lee[2], James C. Sacchettini[1], and Tatyana I. Igumenova[1*]

[1]Department of Biochemistry and Biophysics, Texas A&M University, TX77840, U.S.A.

[2]College of Pharmacy, Seoul National University, Seoul 08826, Republic of Korea

*Correspondence to: Tatyana.Igumenova@ag.tamu.edu

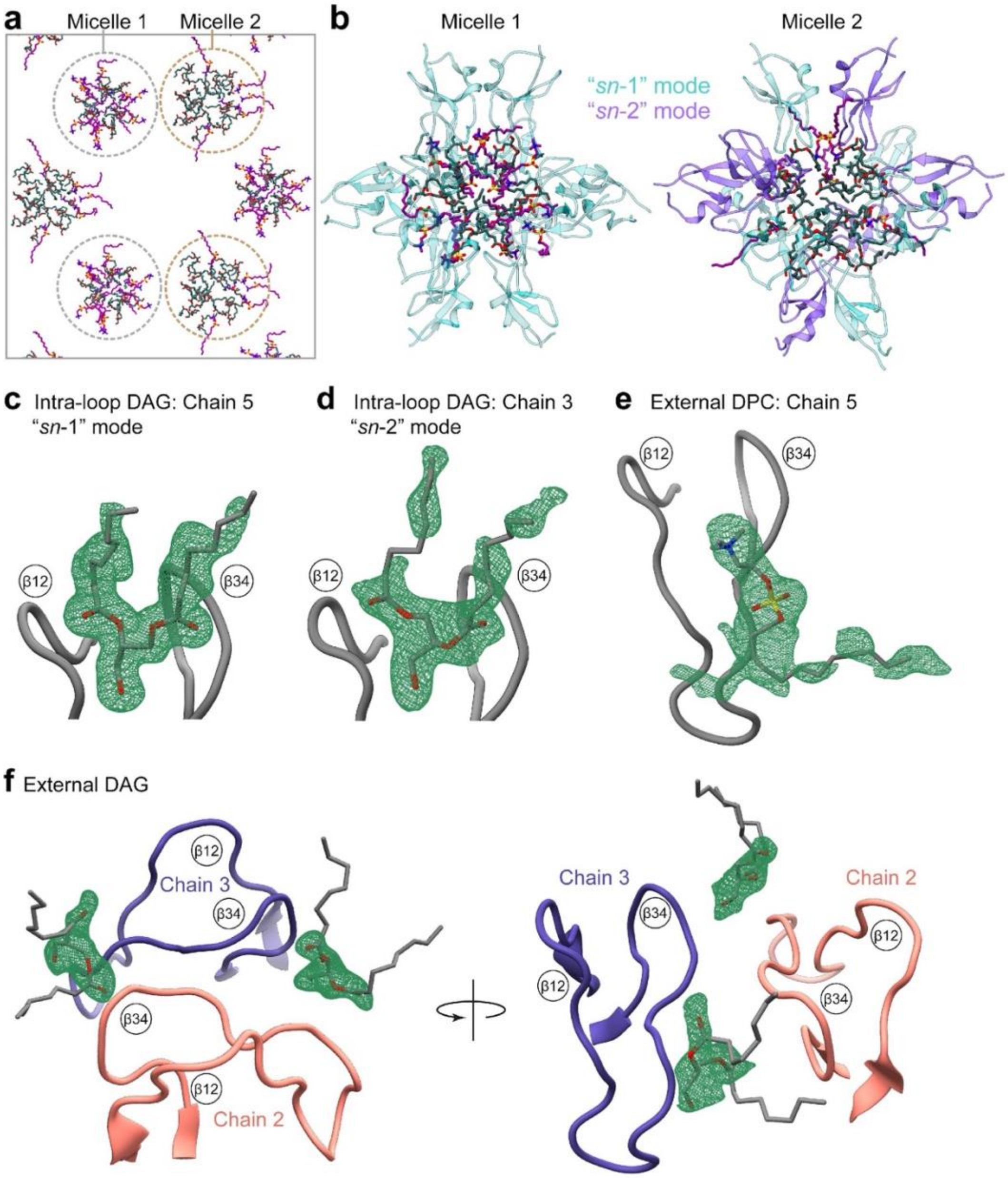

Supplementary Fig. 1 Organization of DAG and DPC molecules in the crystal. a The DAG and DPC molecules are ordered within the crystal lattice as two mixed micelles, micelle 1 (12 DAG and 12 DPC molecules) and micelle 2 (18 DAG and 6 DPC molecules). **b** The C1Bδ chains are arranged with their lipid-sensing loops directly binding to DAG within micelles. Micelle 1 supports only "*sn-1*" binding mode, while micelle 2 supports both, "*sn-1*" and "*sn-2*". **c**-**f** Representative $2F_0$-F_c Polder omit electron density maps of DAG and DPC molecules. **c** and **d** The $2F_0$-F_c electron density maps of DAG molecules bound in the "*sn*-1" (chain 5, contoured at 2.7σ) and "*sn*-2" (chain 3, contoured at 2.8σ) modes within the intra-loop groove of C1Bδ. **e** The $2F_0$-F_c electron density map, contoured at 2.8σ, of the DPC bound externally to chain 5 of C1Bδ. **f** The $2F_0$-F_c electron density maps, each contoured at 2.5σ, of two DAG molecules bound externally to C1Bδ chains 2 and 3.

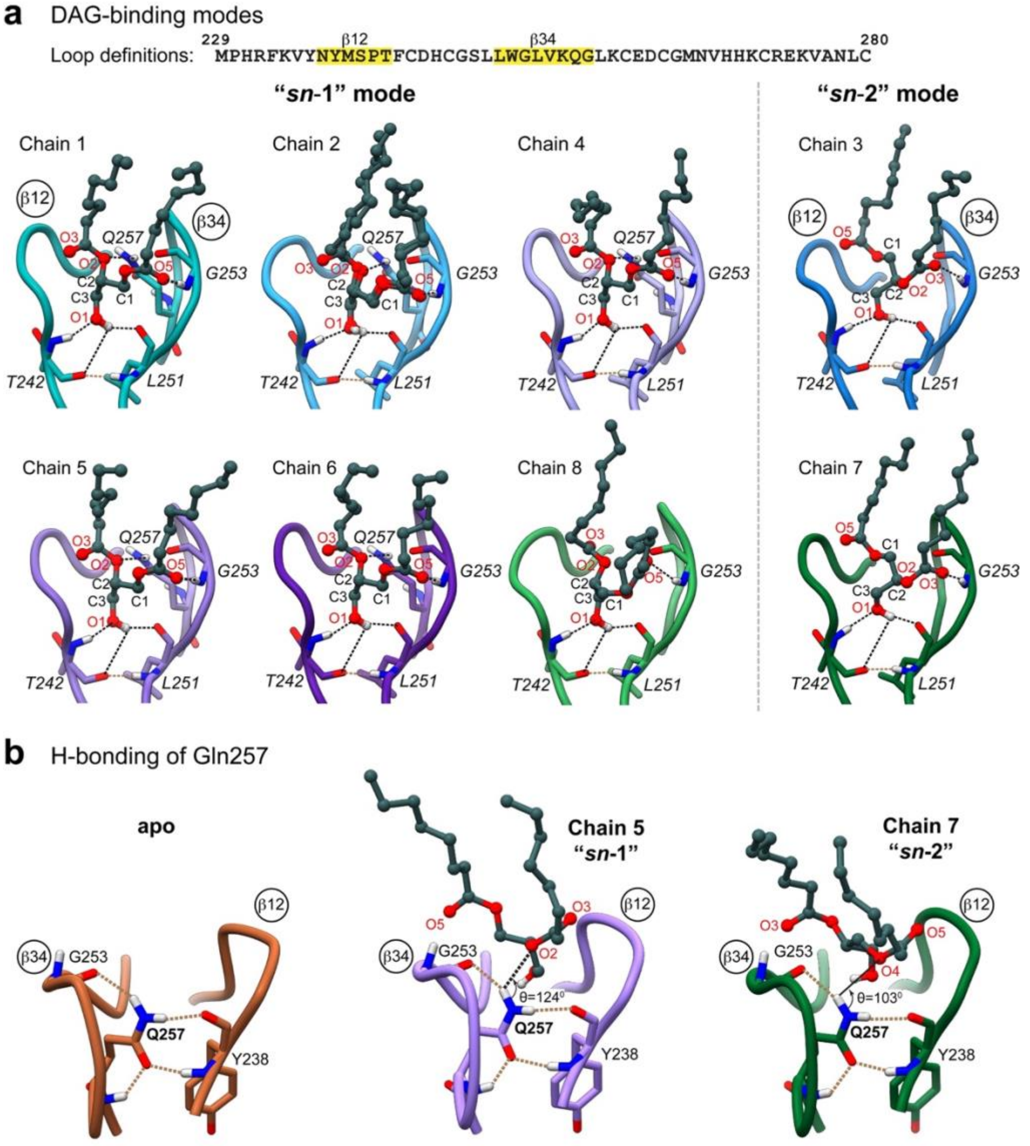

Supplementary Fig. 2 DAG binding modes and hydrogen-bonding patterns for the C1Bδ protein chains in the asymmetric unit. **a** 6 and 2 C1Bδ protein chains have DAG bound in the “*sn*-1” and “*sn*-2” modes, respectively. In the “*sn*-1” mode, the H-bond acceptor of Gly253 amide hydrogen is the carbonyl oxygen of the *sn*-1 ester group (O5), whereas in the “*sn*-2” mode the acceptor is the carbonyl oxygen of the *sn*-2 ester group (O3). The sidechain of Gln257 makes a hydrogen bond with the alkoxy O2 oxygen of DAG in 5 out of 6 “*sn*-1” complexes. **b** Gln257 stabilizes the intra-loop region by forming four hydrogen bonds (brown dashed lines) that stitch the β12 and β34 loops together. These bonds are present in the apo and all DAG-complexed C1Bδ structures. The hydrogens of the Gln257 sidechain amide group also serve as the H-bond donors to the DAG alkoxy oxygen O2 in the “*sn*-1” mode (illustrated using Chain 5, black dashed line). The equivalent interaction in the “*sn*-2” mode involving the alkoxy O4 oxygen would have a non-optimal angle and therefore is unlikely to occur.

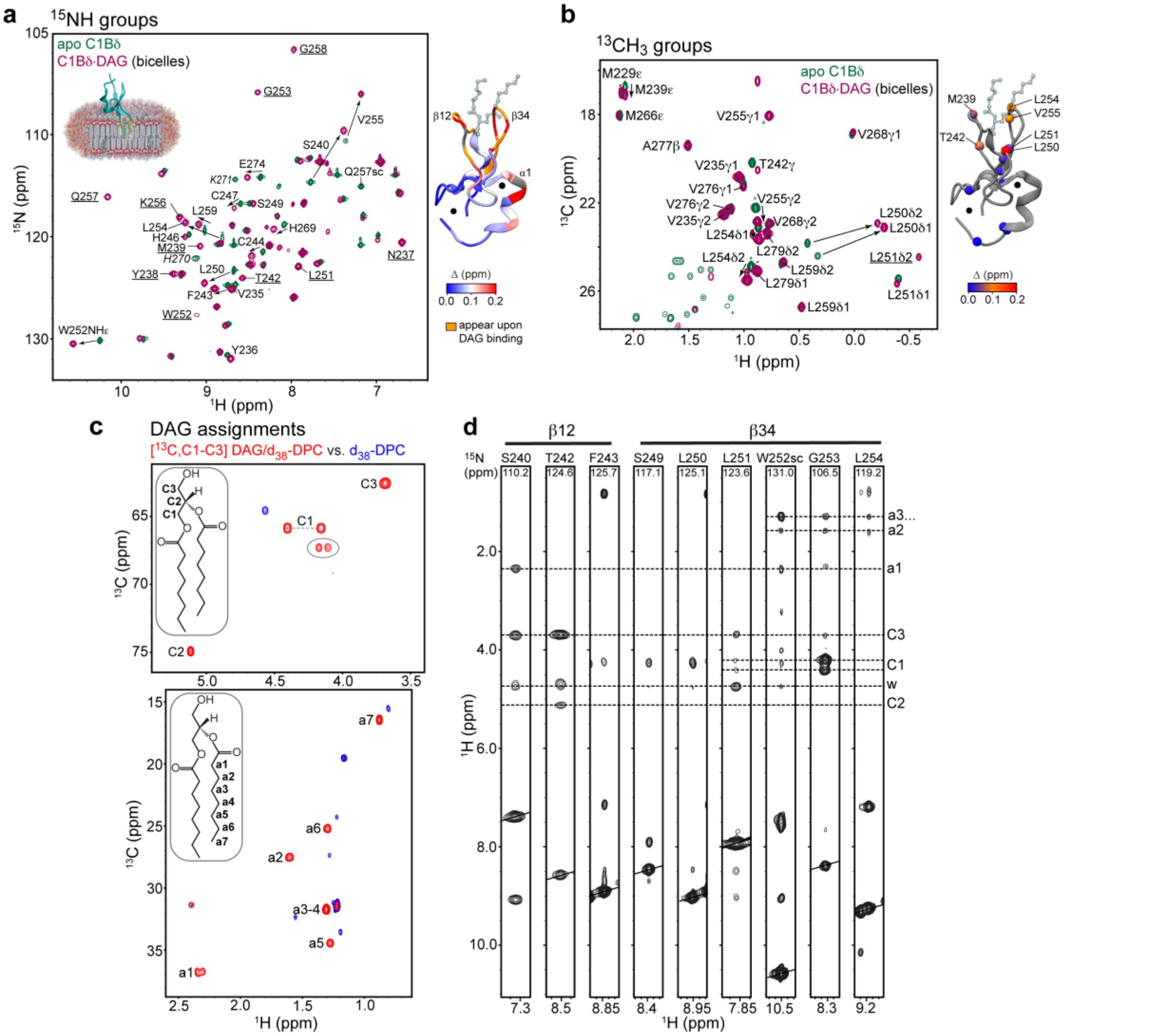

Supplementary Fig. 3 Formation of the ternary C1Bδ-DAG-bicelle complex detected by solution NMR spectroscopy. Overlays of the ^{15}N-^{1}H (**a**) and the methyl ^{13}C-^{1}H HSQC spectra (**b**) of apo C1Bδ (green) and DAG-bound complex in bicelles (maroon). The formation of the C1Bδ-DAG-bicelle complex is evidenced by: (i) the chemical shift changes of the amide and methyl peaks mapped onto the 3D structure of C1Bδ (right side of panels **a** and **b**), and (ii) the reappearance, upon DAG binding, of the amide cross-peaks (underlined, and mapped on 3D structure in orange) that were exchange-broadened in apo C1Bδ. In (**a**), two residues that belong to the C-terminal α-helix, K271 and H270 (italicized), broaden upon association with bicelles and their cross-peaks are not visible at this contour threshold. **c** Atom-specific assignments of the ^{13}C and ^{1}H resonances of DAG mapped onto the ^{13}C-^{1}H HSQC spectra of DAG embedded into the deuterated DPC micelles (red). Circled cross-peaks belong to the *sn*-1,3 isomer. The spectrum of residual ^{1}H in deuterated DPC (blue) is shown as an overlay for comparison. **d** ^{1}H-$^{1}H_N$ strips from the 3D ^{15}N-edited NOESY-TROSY spectrum obtained for the C1Bδ-DAG-bicelle complex. The NH groups of loop residues show inter-molecular NOEs to the ^{1}H atoms of the DAG glycerol moiety that are consistent with the "*sn*-1" binding mode. Also present are the NOEs to the methylene ^{1}H that belong to the acyl groups of DAG or bicelle lipids. "w" denotes water protons. Intra-protein ^{1}H-^{1}H NOEs are suppressed by protein deuteration.

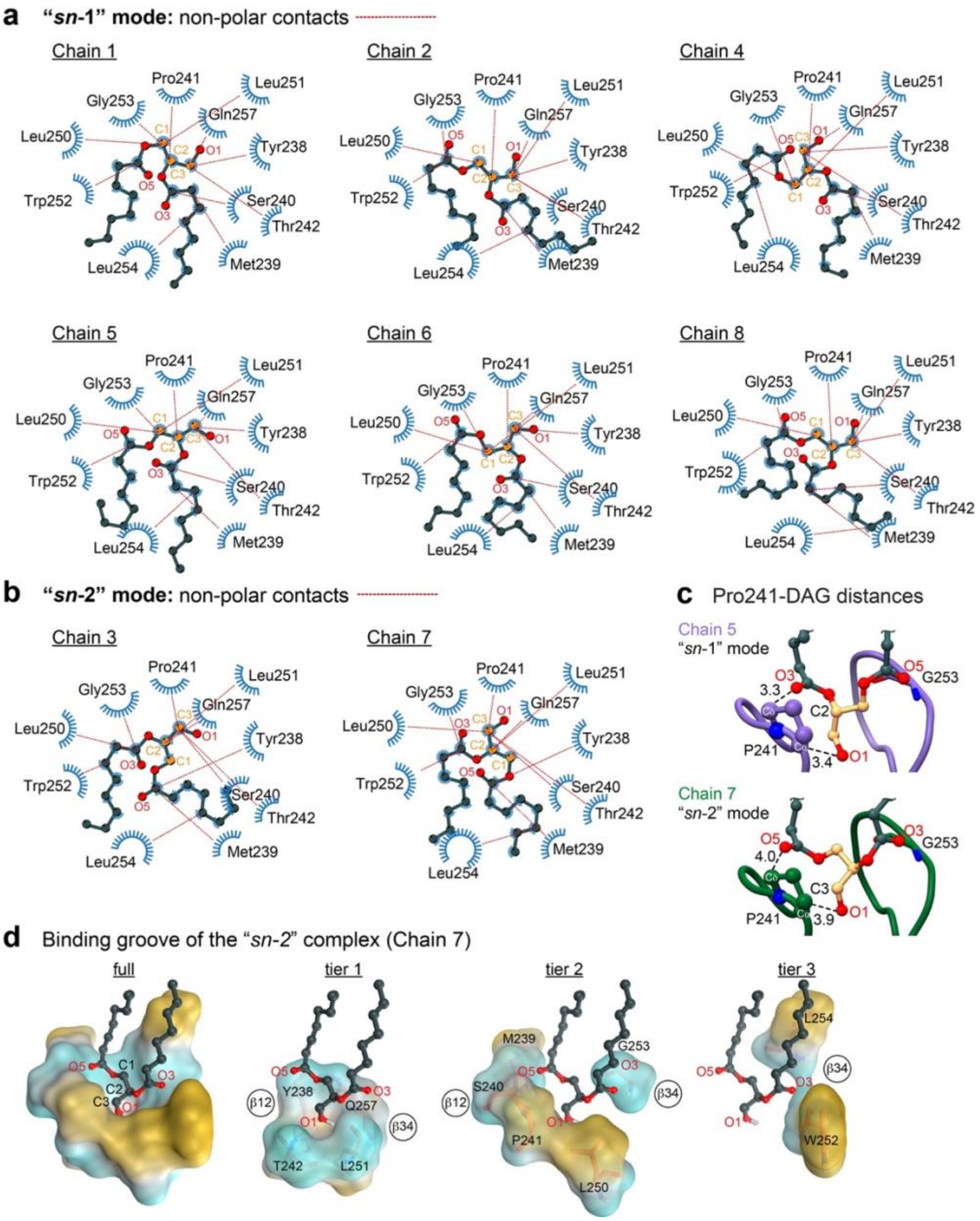

Supplementary Fig. 4 Nonpolar C1Bδ-DAG contacts and the properties of the DAG-binding groove in the “*sn*-2” complex. **a** and **b** 2D LigPlot+ diagrams of nonpolar C1Bδ-DAG interactions. The contact cutoff for backbone and sidechain carbon atoms is 4.5 Å. A subset of contacts is shown with lines to guide the eye. **c** Pro241 makes non-polar contacts with C2 (“*sn*-1” mode) or C3 (“*sn*-2” mode) of DAG. In addition, pyrrolidine Cδ/Cα are within optimal distance from DAG oxygens in both modes to support C-H…O interactions (denoted by dashed lines with distances) **d** Polar backbone atoms and hydrophobic sidechains of DAG-interacting C1Bδ residues create a binding site whose properties are tailored to capture the amphiphilic DAG molecule. This is illustrated through the deconstruction of the binding site into three tiers that accommodate the glycerol backbone (tier 1), the *sn*-1/2 ester groups (tier 2), and the acyl chain methylenes (tier 3).

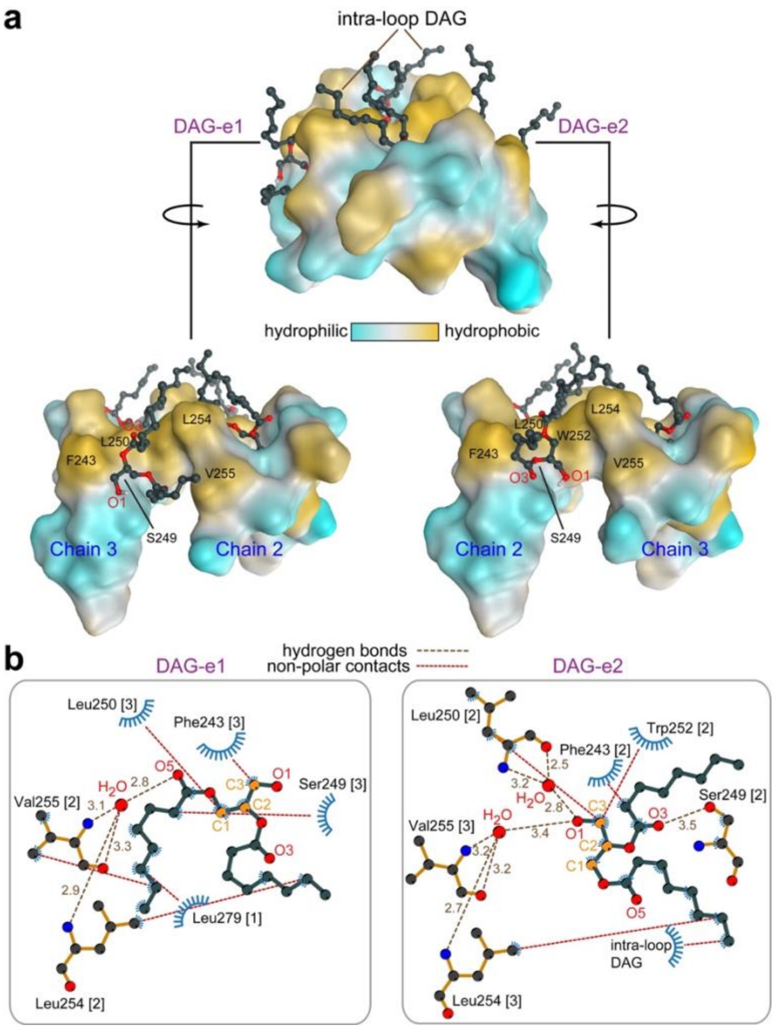

Supplementary Fig. 5 Peripheral interactions of DAG with C1Bδ. **a** Two DAG molecules, DAG-e1 and DAG-e2, peripherally bind to the external hydrophobic grooves created by the C1Bδ chains 2 and 3. **b** The peripheral C1Bδ-DAG interactions involve hydrogen bonds and non-polar contacts, as shown in the 2D $LigPlot^+$ interaction diagrams. Most hydrogen bonds are mediated by water molecules (red spheres) that bridge the polar groups of external DAG molecules to the polar backbone atoms of Val255, Leu254, and Leu250. Non-polar contacts involve hydrophobic residues Phe243, Leu250, Trp252, Leu254, and Val255. The protein chain number is given in square brackets.

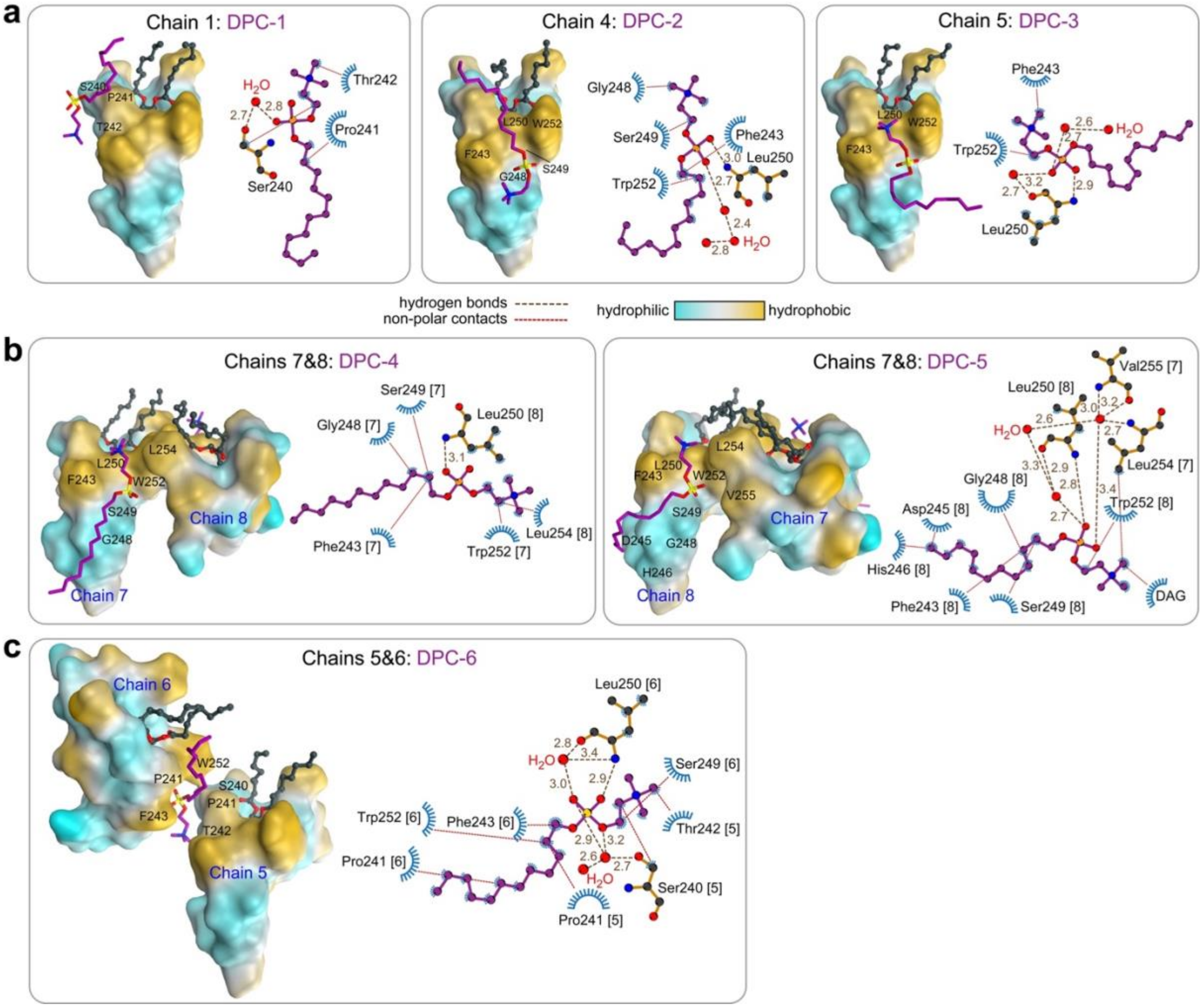

Supplementary Fig. 6 Peripheral interactions of DPC with C1Bδ. The 8-chain asymmetric unit contains six DPC molecules that peripherally interact with C1Bδ. **a** DPC-1, -2, and -3 associate with individual C1Bδ chains 1, 4, and 5. **b** DPC-4 and DPC-5 bind in the grooves created by chains 7 and 8. **c** DPC-6 is wedged between chains 5 and 6. 2D interaction diagrams between C1Bδ and DPC reveal several common patterns. The phosphate oxygens of DPCs engage in hydrogen bonds with solvent molecules and the NH group of the Leu250 backbone. The methyls of the phosphocholine group, along with the aliphatic tails of DPC, form non-polar contacts with the hydrophobic residues of the β12 and β34 loops. The loop residues that show most interactions with the DPC molecules are Phe243 of β12, and Leu250 and Trp252 of β34. In (**a**)-(**c**), DPC is shown in purple, DAG in gray, and the surface of the protein chains is color-coded according to its hydrophilicity/hydrophobicity.

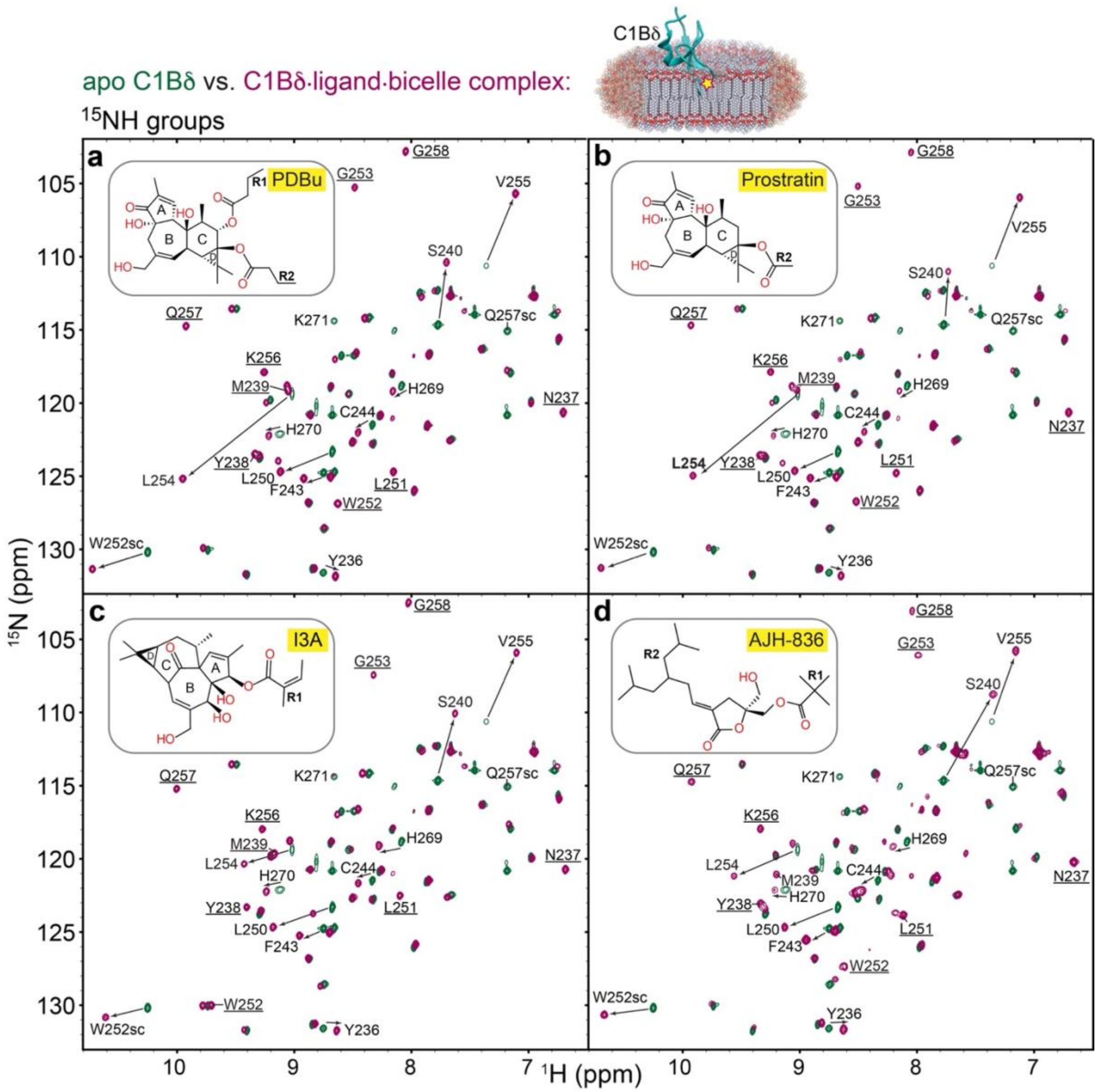

Supplementary Fig. 7 Formation of the ternary C1Bδ-ligand-bicelle complexes detected by solution NMR spectroscopy. Overlays of the ^{15}N-^{1}H HSQC spectra of apo C1Bδ (green) and its complexes (maroon) with **a**, PDBu; **b**, Prostratin; **c**, I3A; and **d**, AJH-836, all in bicelles. The formation of the C1Bδ-ligand-bicelle complexes is evidenced by: (i) the chemical shift changes of the amide peaks, and (ii) the appearance, upon ligand binding, of cross-peaks (underlined) that were exchanged-broadened in apo C1Bδ. The protein-to-ligand ratios were 1:1.2 (PDBu), 1:1.2 (Prostratin), 1:1.2 (I3A), and 1:6 (AJH-836).

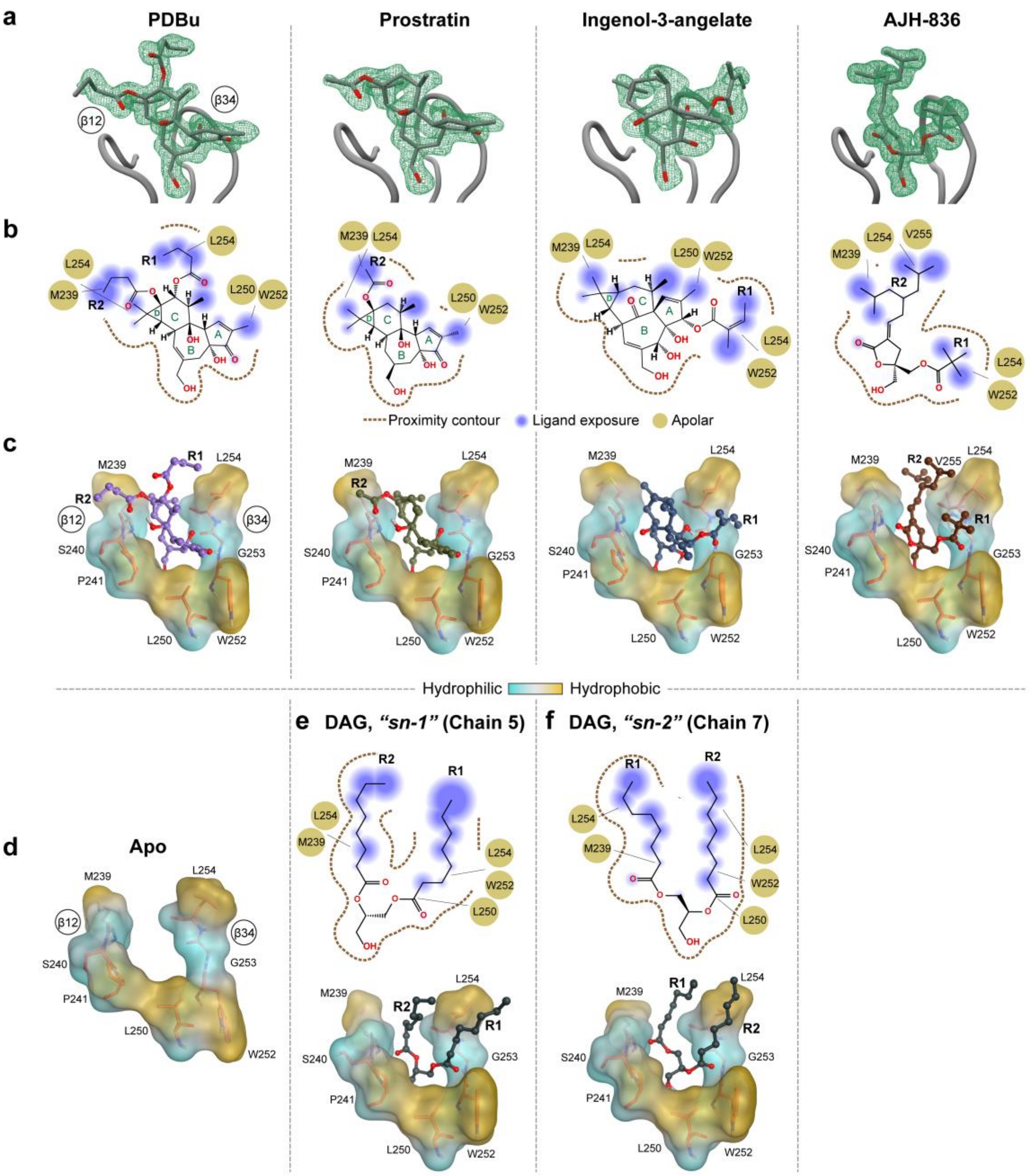

Supplementary Fig. 8 Ligand position and interactions in the groove region of C1Bδ. **a** The $2F_0$-F_c Polder omit electron density maps showing well-resolved electron densities of the ligands in the C1Bδ complexes. The maps were contoured at 2.0σ (PDBu), 2.8σ (prostratin), and 2.3σ (ingenol-3-angelate and AJH-836). **b** 2D diagrams of the ligand placement in the binding groove outlined with the dashed line. The ligand chemical groups that protrude out of the groove are highlighted with blue spheres, with the radius proportional to the degree of solvent exposure. Also shown are protein residues involved in non-polar interactions with the hydrophobic moieties of ligands. **c** 3D representations of the ligand-filled grooves that show the placement of hydrophobic R groups in the complexes. **d** The 3D arrangement of groove-forming residues in apo C1Bδ is presented for comparison. **e,f** 2D diagrams of the DAG placement and 3D representations of the DAG-filled groves in the “*sn-1*” and “*sn-2*” complexes, respectively.

Supplementary Note 1. C1 consensus sequence and the role of individual C1 residues

In addition to the two Zn^{2+}-coordinating Cys_3His motifs, DAG-sensitive C1 domains share a well-defined consensus amino acid sequence[1]. This consensus signature suggests that sidechain-specific interactions are essential for binding the membrane-embedded ligands. Yet, with the notable exception of DAG interactions with the Gln257 sidechain in the "*sn*-1" mode C1B-DAG complex, none of the ligands engage in sidechain-specific hydrogen bonds with protein residues. All H-bonding interactions are mediated exclusively by the polar backbone atoms of residues Thr242, Leu251, and Gly253. Our structures of multiple C1Bδ-ligand complexes now reveal the specific roles of conserved residues in those interactions for the first time. In doing so, this information not only provides a functional rationale for the consensus sequence of DAG-sensitive C1 domains, but it also explains the outcomes of extensive C1/PKC mutagenesis experiments conducted by multiple groups[2-6].

The four strictly conserved non-Zn^{2+} coordinating residues are Pro241, Gly253, and the Gln257-Gly258 forming the "QG" motif (Fig. 6f, red). Pro241 anchors the carbonyl-containing moieties of DAG and exogenous agonists to the β12 loop (Fig. 6b-e and Supplementary Fig. 4c). In addition to stereospecific H-bonding interactions with the ligands (*vide supra*), Gly253 forms the "depression" in the loop β34 surface that accommodates the hydrophobic moieties of DAG (Fig. 2b,c), of the exogenous ligands (Supplementary Fig. 8c), and potentially of the lipids (Fig. 5c) to create a continuous hydrophobic surface. Gln257 stabilizes the loop region through the formation of inter-loop hydrogen bonds and, in the case of DAG, makes a hydrogen bond with the ligand (Fig. 2b and Supplementary Fig. 2). The subsequent glycine residue, Gly258, ensures the conformational flexibility of the β34 loop hinge[7, 8] that likely facilitates the membrane recruitment.

Of the nine conserved hydrophobic residues, six form the hydrophobic protein core (Fig. 6f) in the structural Zn^{2+} region (Phe233, Met266, Val268, and Val276) and the inter-loop (Tyr238 and Leu251) regions. The sidechains of the other three (Leu250, Trp252, and Leu254), together with strictly conserved Pro241 and the consensus aromatic residue Phe243, form the outside hydrophobic "cage" that

envelopes the bound ligands (Fig. 6g,h). One interesting feature of the "cage" is that, along with the expected inter-residue hydrophobic contacts, there is an opportunity for Leu250 to engage in CH-π interactions[9] with the aromatic rings of Trp252 and Phe243. Overall, the spatial organization of these five residues enables the loop region of C1B to effectively interface with lipids, while "shielding" the hydrophilic ligand moieties from the hydrophobic membrane environment (Fig. 2b,c; Fig. 5). Of note, in the complex of C1Bδ with a water-soluble hydrophilic phorbol ester crystallized in the absence of lipids/detergents, the Trp252 sidechain is oriented away from the membrane-binding loop region (Fig. 6i), as is observed in apo C1Bδ (Fig. 2a). The support for Trp252 reorientation upon formation of the ternary C1-ligand-membrane complex in solution comes from the unusually large upfield chemical shift change experienced by the Leu250 methyl groups (Fig. 2e, inset). This shift is consistent with the aromatic ring current effect, induced by the Trp252 sidechain being brought into close proximity to the Leu250 methyl groups upon DAG binding.

Supplementary Note 2. C1 domain interactions with lipids: implications for differential DAG affinities of PKC isoforms.

The packing arrangements of the C1Bδ chains in the crystal, and their amphiphilic surfaces, create opportunistic binding sites for external DAG and membrane-mimicking agents. There are two peripheral DAG molecules anchored to the groove formed between C1Bδ chains 2 and 3 via hydrogen bonds and nonpolar contacts (Supplementary Fig. 5). The hydrogen-bonding interactions are mediated by water molecules that bridge DAG oxygen atoms and the polar backbone atoms of Leu250, Leu254, and Val255. The sidechains of these three residues, along with those of Phe243 and Trp252, engage in nonpolar interactions with DAG acyl chains. In addition to peripheral DAG, six DPC molecules engage in non-polar and water-mediated polar contacts with the C1Bδ loop region surface (Supplementary Fig. 6). We observed similar peripheral association of DHPC with protein in the C1Bδ-exogenous agonist complexes (Fig. 4).

The lipophilic properties of Trp252 at the "DAG-toggling" position are directly relevant to the question of DAG sensitivity of PKC isoforms. Indeed, the identity of an aromatic residue at position 252 or equivalent has a profound effect on the C1B DAG affinity and hence the DAG sensitivity of the parent PKC[5, 7, 8, 10, 11]. The novel (Ca^{2+}-independent) PKC isoforms have a Trp at that position and show high DAG affinities, while the C1B of conventional (Ca^{2+}-dependent) isoforms have a Tyr and show lower DAG affinities. It is suggested that the higher DAG affinities of novel PKC isoform C1B domains compensate for the lack of Ca^{2+}-dependent C2 domains that co-target conventional PKCs to anionic membranes[11].

The interaction pattern of Trp252 with ligands (Fig. 2b,c; Fig. 3a; Fig. 5b,c) and its membrane insertion (Fig. 3b) now makes it possible to rationalize why having Trp over Tyr is thermodynamically advantageous for C1B domains. Compared to the Tyr sidechain, Trp has higher hydrophobicity[12], aromaticity[13, 14], and larger electric dipole moment[15]. The hydrophobicity factor is relevant in membrane partitioning[5], and in engaging both "cage" residues (Fig. 6g) and ligands (Supplementary Fig. 4a,b; Supplementary Fig. 8b,c). The extended aromatic system with two fused rings enables cation-π[13] and CH-π[9] interactions that are observed even in the crystalline state in the absence of a fully formed membrane environment (Figs. 4f and 6g,h). In addition, the ~two-fold larger electric dipole moment of the Trp sidechain likely facilitates charge-dipole and dipole-dipole interactions in the complex electrostatic environment of the membrane headgroup region and its interfacial water molecules.

Supplementary Note 3. C1-DAG lactone interactions

The development of DAG lactones for the modulation of PKC activity was pioneered by the Blumberg and Marquez laboratories[16-25]. The concept behind the development of this class of compounds is that, compared to the endogenous ligand DAG, the rigid cyclic lactone structure reduces the entropic penalty for binding lactones to C1 domains. Furthermore, the hydrophobicity of DAG lactones can be varied via *sn-1/2* linked substituents R1 and R2. Combinatorial library built upon the lactone template identified the R1/R2 substituent combinations that produced distinct and specific cellular responses,

attesting to the promise of this class of compounds as modulators of DAG effector proteins[26]. The lactone AJH-836[27] used for our structural studies has one of the highest reported affinities among this class of compounds, and AJH-836 shows selectivity towards the novel PKC isozymes δ and ε relative to conventional α and βII[16].

DAG lactones can bind to the C1 domains in two orientations: (i) "*sn-1*", where the carbonyl of the *sn*-1 ester group hydrogen-bonds with the amide N-H group of Gly253, and (ii) "*sn-2*", where the carbonyl oxygen of the lactone ring hydrogen-bonds with the N-H of Gly253. Previous docking studies, conducted using the Phorbol 13-monoacetate complexed C1Bδ structure[28] suggested that the "*sn*-2" binding mode is preferred for the DAG lactones[29]. Notably, the Trp252 sidechain is oriented away from the membrane-binding loops in that C1Bδ structure (Fig. 6i). Our structure of the C1Bδ-AJH-836 complex, where the Trp252 sidechain is oriented towards the membrane-binding region, has the ligand bound in the "*sn*-1" mode (Fig. 5). The preference for the "*sn*-1" mode likely originates from the favorable positioning of bulky R1 and R2 groups relative to the hydrophobic sidechains of the rim residues. Specifically, R1 is sandwiched between Leu254 and Trp252, and R2 contacts Met239, Leu254, and Val255. This arrangement maximizes the protein-ligand hydrophobic contacts and creates a contiguous lipophilic surface for membrane interactions. In this context, the significance of the Trp252 sidechain reorientation (Fig. 2a) upon complexation with PKC agonists becomes clear. When oriented towards the loop region, Trp252 displays considerable functional versatility as it can interface simultaneously with the hydrophobic ligand and the surrounding lipids (*vide supra*).

Supplementary Table 1 Crystallography data collection, and refinement statistics of apo C1Bδ and its complexes with diacylglycerol and AJH-836.

	**Apo (7KND)**	**Diacylglycerol (7L92)**	**AJH 836-DHPC (7LEO)**	**AJH 836 (7LF3)**
**Data Collection**				
Space group	P 41	H 3	C 1 2 1	C 1 2 1
Cell dimensions				
a, *b*, *c* (Å)	37.26, 37.26, 31.89	89.07, 89.07, 218.68	83.39, 50.89, 37.48	34.91, 25.94, 57.64
α, *β*, *γ* (°)	90.00, 90.00, 90.00	90.00, 90.00, 120.00	90.00, 107.7 90.00	90.00, 92.57, 90.00
Resolution (Å)	37.26–1.39 (1.41-1.39)	44.60 – 1.75 (1.78-1.75)	50 – 1.65 (1.68 – 1.65)	28.79 – 1.13 (1.15 – 1.13)
R_{merge}	0.056 (1.65)	0.07 (0.613)	0.1 (0.831)	0.13 (2.3)
$I/\sigma(I)$	15 (0.8)	6.8 (1.6)	7.4 (1.77)	15.5 (0.6)
Completeness (%)	100 (100)	99.1 (99.6)	98.5 (98.1)	95.6 (86.6)
Redundancy	6.3 (4.3)	2.8 (2.8)	5.4 (3.7)	5.7 (5.5)
CC1/2	0.995 (0.522)		0.99 (0.568)	0.98 (0.396)
**Refinement**				
Resolution (Å)	37.26 – 1.39	28.9-1.75	29.96 – 1.65	28.79 – 1.13
No. reflections	8897	64618	17697	18562
R_{work} / R_{free}	0.216 / 0.246	0.2142 / 0.2460	0.2237 / 0.2439	0.1826 /0.1993
No. atoms				
Protein	422	3371	819	440
Solvent	26	231	61	51
Ligands/metals	2	400	95	62
B factors ($Å^2$)				
Protein	31	36	33	18
Ligands/metals	25	39	38.3	31.5
R.m.s. deviations				
Bond lengths (Å)	0.01	0.009	0.006	0.006
Bond angles (°)	1.35	1.27	1.1	0.942

Supplementary Table 2 Crystallography data collection, and refinement statistics of the C1Bδ complexes with PDBu, Prostratin, and Ingenol-3-angelate.

	**Phorbol 12,13-dibutyrate (7KNJ)**	**Prostratin (7LCB)**	**Ingenol-3-angelate (7KO6)**
**Data Collection**			
Space group	C 1 2 1	C 1 2 1	C 1 2 1
Cell dimensions			
a, *b*, *c* (Å)	34.20, 26.14, 58.16	34.40, 25.87, 57.96	34.38, 25.91, 57.73
α, *β*, *γ* (°)	90.00, 96.92, 90.00	90.00, 97.29, 90.00	90.00, 98.39, 90.00
Resolution (Å)	57.73–1.57 (1.67-1.57)	57.50 – 1.70 (1.8 – 1.7)	57.11 – 1.80 (1.90 - 1.80)
R_{merge}	0.055 (0.2054)	0.06 (0.2636)	0.1144 (0.4800)
$I/\sigma(I)$	21.05 (3.28)	15.48 (3.00)	15.76 (3.79)
Completeness (%)	85.1 (34)	89.1 (48.4)	100 (100.0)
Redundancy	5.71 (0.44)	3.85 (0.63)	12.54 (8.97)
CC1/2			
**Refinement**			
Resolution (Å)	19.24-1.57	28.75 - 1.7	28.56-1.8
No. reflections	6194	5113	4815
R_{work} / R_{free}	0.1772 / 0.2236	0.1719 / 0.2136	0.1757 / 0.2154
No. atoms			
Protein	431	431	423
Solvent	44	38	53
Ligands/metals	97	62	65
B factors (Å^2)			
Protein	13	13	14
Ligands/metals	23.6	20	20
R.m.s. deviations			
Bond lengths (Å)	0.008	0.009	0.01
Bond angles (°)	2.42	1.445	2.19

References

1. Hurley J. H., Newton A. C., Parker P. J., Blumberg P. M., Nishizuka Y. Taxonomy and function of C1 protein kinase C homology domains. *Protein Sci* **6**, 477-480 (1997).

2. Kazanietz M. G., Wang S., Milne G. W., Lewin N. E., Liu H. L., Blumberg P. M. Residues in the second cysteine-rich region of protein kinase C delta relevant to phorbol ester binding as revealed by site-directed mutagenesis. *J Biol Chem* **270**, 21852-21859 (1995).

3. Choi Y., *et al.* Conformationally Constrained Analogues of Diacylglycerol (DAG). 28. DAG-dioxolanones Reveal a New Additional Interaction Site in the C1b Domain of PKCδ. *Journal of Medicinal Chemistry* **50**, 3465-3481 (2007).

4. Rahman G. M., *et al.* Identification of the activator-binding residues in the second cysteine-rich regulatory domain of protein kinase Ctheta (PKCtheta). *Biochem J* **451**, 33-44 (2013).

5. Stewart M. D., Cole T. R., Igumenova T. I. Interfacial partitioning of a loop hinge residue contributes to diacylglycerol affinity of conserved region 1 domains. *J Biol Chem* **289**, 27653-27664 (2014).

6. Wang Q. J., Fang T. W., Nacro K., Marquez V. E., Wang S., Blumberg P. M. Role of hydrophobic residues in the C1b domain of protein kinase C delta on ligand and phospholipid interactions. *J Biol Chem* **276**, 19580-19587 (2001).

7. Stewart M. D., Morgan B., Massi F., Igumenova T. I. Probing the determinants of diacylglycerol binding affinity in the C1B domain of protein kinase Calpha. *J Mol Biol* **408**, 949-970 (2011).

8. Stewart M. D., Igumenova T. I. Toggling of Diacylglycerol Affinity Correlates with Conformational Plasticity in C1 Domains. *Biochemistry* **56**, 2637-2640 (2017).

9. Plevin M. J., Bryce D. L., Boisbouvier J. Direct detection of CH/pi interactions in proteins. *Nat Chem* **2**, 466-471 (2010).

10. Dries D. R., Gallegos L. L., Newton A. C. A single residue in the C1 domain sensitizes novel protein kinase C isoforms to cellular diacylglycerol production. *J Biol Chem* **282**, 826-830 (2007).

11. Giorgione J. R., Lin J. H., McCammon J. A., Newton A. C. Increased membrane affinity of the C1 domain of protein kinase Cdelta compensates for the lack of involvement of its C2 domain in membrane recruitment. *J Biol Chem* **281**, 1660-1669 (2006).

12. Wimley W. C., White S. H. Experimentally determined hydrophobicity scale for proteins at membrane interfaces. *Nat Struct Biol* **3**, 842-848 (1996).

13. Dougherty D. A. Cation-pi interactions involving aromatic amino acids. *J Nutr* **137**, 1504S-1508S; discussion 1516S-1517S (2007).

14. de Jesus A. J., Allen T. W. The role of tryptophan side chains in membrane protein anchoring and hydrophobic mismatch. *Biochim Biophys Acta* **1828**, 864-876 (2013).

15. Yau W. M., Wimley W. C., Gawrisch K., White S. H. The preference of tryptophan for membrane interfaces. *Biochemistry* **37**, 14713-14718 (1998).

16. Cooke M., *et al.* Characterization of AJH-836, a diacylglycerol-lactone with selectivity for novel PKC isozymes. *J Biol Chem* **293**, 8330-8341 (2018).

17. Ohashi N., *et al.* Synthesis and Evaluation of Dimeric Derivatives of Diacylglycerol-Lactones as Protein Kinase C Ligands. *Bioconjug Chem* **28**, 2135-2144 (2017).

18. Nomura W., *et al.* Synthetic caged DAG-lactones for photochemically controlled activation of protein kinase C. *Chembiochem* **12**, 535-539 (2011).

19. Malolanarasimhan K., *et al.* Conformationally constrained analogues of diacylglycerol (DAG). 27. Modulation of membrane translocation of protein kinase C (PKC) isozymes alpha and delta by diacylglycerol lactones (DAG-lactones) containing rigid-rod acyl groups. *J Med Chem* **50**, 962-978 (2007).

20. Pu Y., *et al.* A novel diacylglycerol-lactone shows marked selectivity in vitro among C1 domains of protein kinase C (PKC) isoforms alpha and delta as well as selectivity for RasGRP compared with PKCalpha. *J Biol Chem* **280**, 27329-27338 (2005).

21. Lee J., *et al.* Conformationally constrained diacylglycerol (DAG) analogs: 4-C-hydroxyethyl-5-O-acyl-2,3-dideoxy-D-glyceropentono-1,4-lactone analogs as protein kinase C (PKC) ligands. *Eur J Med Chem* **39**, 69-77 (2004).

22. Choi Y., Kang J. H., Lewin N. E., Blumberg P. M., Lee J., Marquez V. E. Conformationally constrained analogues of diacylglycerol. 19. Synthesis and protein kinase C binding affinity of diacylglycerol lactones bearing an N-hydroxylamide side chain. *J Med Chem* **46**, 2790-2793 (2003).

23. Nacro K., Bienfait B., Lewin N. E., Blumberg P. M., Marquez V. E. Diacylglycerols with lipophilically equivalent branched acyl chains display high affinity for protein kinase C (PK-C). A direct measure of the effect of constraining the glycerol backbone in DAG lactones. *Bioorg Med Chem Lett* **10**, 653-655 (2000).

24. Lee J., *et al.* Conformationally constrained analogues of diacylglycerol. 12. Ultrapotent protein kinase C ligands based on a chiral 4,4-disubstituted heptono-1,4-lactone template. *J Med Chem* **39**, 36-45 (1996).

25. Lee J., Marquez V. E., Blumberg P. M., Krausz K. W., Kazanietz M. G. Conformationally constrained analogues of diacylglycerol (DAG)--II. Differential interaction of delta-lactones and gamma-lactones with protein kinase C (PK-C). *Bioorg Med Chem* **1**, 119-123 (1993).

26. Duan D., *et al.* Conformationally constrained analogues of diacylglycerol. 29. Cells sort diacylglycerol-lactone chemical zip codes to produce diverse and selective biological activities. *J Med Chem* **51**, 5198-5220 (2008).

27. Ann J., *et al.* Design and synthesis of protein kinase C epsilon selective diacylglycerol lactones (DAG-lactones). *Eur J Med Chem* **90**, 332-341 (2015).

28. Zhang G. G., Kazanietz M. G., Blumberg P. M., Hurley J. H. Crystal-Structure of the Cys2 Activator-Binding Domain of Protein-Kinase C-Delta in Complex with Phorbol Ester. *Cell* **81**, 917-924 (1995).

29. Sigano D. M., *et al.* Differential binding modes of diacylglycerol (DAG) and DAG lactones to protein kinase C (PK-C). *J Med Chem* **46**, 1571-1579 (2003).
